# Repurposing drugs to fast-track therapeutic agents for the treatment of cryptococcosis

**DOI:** 10.7717/peerj.4761

**Published:** 2018-05-04

**Authors:** Megan Truong, Leigh G. Monahan, Dee A. Carter, Ian G. Charles

**Affiliations:** 1 The ithree institute, University of Technology Sydney, Sydney, NSW, Australia; 2 School of Life and Environmental Sciences and the Marie Bashir Institute for Infectious Diseases and Biosecurity, University of Sydney, Sydney, NSW, Australia; 3 Quadram Institute Bioscience, Norwich Research Park, Norwich, United Kingdom

**Keywords:** Cryptococcus, Cryptococcosis, Drug repurposing, Antifungal drugs, Flubendazole, Calcium channel blockers, Drug screening, Benzimidazoles

## Abstract

Many infectious diseases disproportionately affect people in the developing world. Cryptococcal meningitis is one of the most common mycoses in HIV-AIDS patients, with the highest burden of disease in sub-Saharan Africa. Current best treatment regimens still result in unacceptably high mortality rates, and more effective antifungal agents are needed urgently. Drug development is hampered by the difficulty of developing effective antifungal agents that are not also toxic to human cells, and by a reluctance among pharmaceutical companies to invest in drugs that cannot guarantee a high financial return. Drug repurposing, where existing drugs are screened for alternative activities, is becoming an attractive approach in antimicrobial discovery programs, and various compound libraries are now commercially available. As these drugs have already undergone extensive optimisation and passed regulatory hurdles this can fast-track their progress to market for new uses. This study screened the Screen-Well Enzo library of 640 compounds for candidates that phenotypically inhibited the growth of *Cryptococcus deuterogattii*. The anthelminthic agent flubendazole, and L-type calcium channel blockers nifedipine, nisoldipine and felodipine, appeared particularly promising and were tested in additional strains and species. Flubendazole was very active against all pathogenic *Cryptococcus* species, with minimum inhibitory concentrations of 0.039–0.156 μg/mL, and was equally effective against isolates that were resistant to fluconazole. While nifedipine, nisoldipine and felodipine all inhibited *Cryptococcus*, nisoldipine was also effective against *Candida, Saccharomyces* and *Aspergillus*. This study validates repurposing as a rapid approach for finding new agents to treat neglected infectious diseases.

## Introduction

Cryptococcal meningitis is a devastating fungal disease that disproportionately affects the poorest, most resource-limited, underdeveloped regions of the world, where the health burden is extremely high. Those who are most at risk are patients who are immunocompromised, including HIV/AIDS populations and patients receiving rigorous immunosuppressive treatments such as chemotherapy. Mortality rates are unacceptably high and vary significantly with demography, with estimates as low as 9% among patients in developed countries compared to 70% in sub-Saharan Africa (Park et al., 2009), where cryptococcal meningitis clinically presents in up to 40% of HIV/AIDS patients (Dzoyem et al., 2012; Rajasingham et al., 2017). This high mortality is largely attributable to lack of access and difficulty in administering the most effective standard treatment, amphotericin B combined with flucytosine (Kneale et al., 2016; Perfect et al., 2010), which is expensive and requires medical infrastructure for therapeutic monitoring due to adverse effects including nephrotoxicity, hepatotoxicity and bone marrow suppression (Kneale et al., 2016; Loyse et al., 2013; Meiring et al., 2016). Fluconazole is commonly used in place of amphotericin B and flucytosine; however, as it is a fungistatic drug, it does not effectively clear fungal burden and is associated with clinical relapse, greater mortality and poorer clinical outcomes, as well as the risk of inducing drug resistance (Bicanic et al., 2006; Jarvis et al., 2014; Smith et al., 2015). Overall, there is an urgent need for new antifungal treatments that are more effective, economical, accessible and available to regions where they are needed most. Drug repurposing, or repositioning, is becoming an increasingly popular avenue in drug discovery. This is the establishment of a new indication for a drug entity that has been previously approved for another purpose by authoritative bodies such as the US Food and Drug Administration (FDA). For pharmaceutical industries and drug developers, repurposing offers attractive benefits as previously established pharmacokinetic and pharmacodynamics profiles provide the potential to fast-track drugs through the development pipeline, avoiding substantial costs associated with expensive clinical trials (Oprea & Mestres, 2012). The antidepressant sertraline is a leading example of a non-antifungal drug in the process of being repurposed for cryptococcal meningitis. First discovered through a drug screen in 2010, sertraline has potent fungicidal activity against *Cryptococcus* and is synergistic in combination with fluconazole (Zhai et al., 2010, 2012). In vivo, sertraline alone or in combination with fluconazole reduced fungal burden in infected mice (Zhai et al., 2012). Most importantly, sertraline achieved more efficient clearance of fungal burden in HIV-infected patients in Uganda, with no relapse during the clinical study (Rhein et al., 2016).

*Cryptococcus* encompasses a range of phylogenetically discrete genotypes and species that can cause cryptococcal meningitis. *Cryptococcus neoformans* is an opportunistic pathogen commonly infecting groups with compromised immune systems. *C. neoformans* is the most well-studied cryptococcal species and has been the main target of drug discovery efforts to identify new anti-cryptococcal agents. However, *C. deuterogattii* (formerly known as *C. gattii* genotype VGII) has recently emerged as a primary pathogen, responsible for recent outbreaks affecting otherwise immunocompetent individuals (Byrnes et al., 2010; Hagen et al., 2015). In this study, we phenotypically screened the Enzo library consisting of 640 FDA-approved agents against *C. deuterogattii* to identify compounds with novel anti-cryptococcal activity. We report multiple compounds that inhibited the growth of *C. deuterogattii* and that were subsequently screened against various pathogenic fungal species to determine the spectrum of activity. Flubendazole was the most potent compound and was found to specifically target *Cryptococcus* species. Nifedipine, nisoldipine and felodipine demonstrated antifungal activity towards *Cryptococcus* species and to varying degrees towards other yeast and mould pathogens. Our findings highlight the value of repurposing strategies to rapidly screen drug libraries that can reveal new classes of antifungal agents for further development.

## Methods

### Strains

Fungal strains used in this study are listed in Tables 1 and 2. Strains were maintained as glycerol stocks stored at −80 °C. Strains were grown on sabouraud dextrose agar (SDA) for 48 h at 30 °C before use.

**Table 1:** Minimum inhibitory concentrations of flubendazole and a series of calcium channel blockers against *Cryptococcus*, *Saccharomyces*, *Candida* and *Aspergillus* species.

Genus	Species	Strain	MIC (μg/mL)^*a*^				
			FLB	NIF	NIG	FELO	NISO
*Cryptococcus*	*deuterogattii*	R265	0.078	10	>40	10–20	5–20
	*gattii*	WM179	0.078	5	>40	10	10–40
	*tetragattii*	M391	0.078	10	>40	≥20	40
	*neoformans*	H99	0.078	10	>40	10	20–40
*Saccharomyces*	*cerevisiae*	S288c	>40	≥40	>40	>40	10
*Candida*	*parapsilosis*	ATCC22019	>40	>40	>40	>40	20
	*albicans*	M23	>40	>40	>40	>40	40
	*glabrata*	M27	>40	20	>40	>40	10
	*glabrata*	M63	>40	20	>40	>40	10
*Aspergillus*	*fumigatus*	ATCC204305	>40	>40	>40	>40	10–20

**Notes:**

MIC ranges are given for compounds with variable results. MICs greater than 40 μg/mL (shaded in grey) indicate where an MIC was not obtained at the highest testing concentration.

FLB, flubendazole; NIF, nifedipine; NIG, niguldipine; FELO, felodipine; NISO, nisoldipine.

a MICs obtained from four biological replicates each with technical duplicates.

**Table 2:** Minimum inhibitory and minimum fungicidal concentrations of flubendazole against *Cryptococcus* species from varied sources.

Species (molecular genotype)	Strain	Source	Geographic origin^a^	MIC (μg/mL)		MFC (μg/mL)
				FLC^b^	FLB^c^	FLB^c^
*C. gattii* (VGI)	ENV316	Environmental	Australia		0.078	0.156
	PNG14	Clinical	Papua New Guinea		0.078	0.078
	2005/215	Clinical	France		0.078	0.078
	V15/571_103	Veterinary	Australia		0.078	0.078
*C. deuterogattii* (VGII)	97/170	Clinical	France	64	0.078	0.078
	R265	Clinical	Canada	4	0.078	0.078
	CBS1930	Veterinary	Aruba		0.078	0.078
	ICB184	Environmental	Brazil		0.039	0.039
	14.1431	Veterinary	Australia	32	0.039	0.078
	LA499	Clinical	Columbia	32	0.039	0.039
	V5	Veterinary	Australia	8	0.039	0.078
	03-201073	Clinical	Australia	16	0.078	0.078
*C. bacillisporus* (VGIII)	VBP62270	Veterinary	Australia		0.078	0.078
	97/427	Clinical	Mexico		0.156	0.313
	WM161	Environmental	USA		0.156	0.156
	B13C	Clinical	Asia		0.078	0.156
*C. tetragattii* (VGIV)	MMRL3013	Clinical	Africa		0.039–0.156	0.039–0.156
	M250	Clinical	Malawi		0.156	0.156
	WM779	Veterinary	South Africa		0.078	0.078
	MMRL2650	Clinical	India		0.039	0.078
*C. neoformans* (VNI)	1043.ENR.STOR	Clinical	Africa		0.078	0.156
	1020.CLIN1	Clinical	Africa		0.039	0.078
	H99	Clinical	USA	4	0.039	0.039
	WM625	Clinical	Australia	8	0.02	0.039
	WM385	Clinical	Thailand	4	0.039	0.039
*C. neoformans* (VNII)	1023.ENR	Clinical	Africa		0.039	0.078
	1045.ENR.STOR	Clinical	Africa		0.078	0.156
*C. neoformans* (VNBI)	1050.ENR.CLIN	Clinical	Africa		0.078	0.156
*C. neoformans* (VNBII)	1033.ENR	Clinical	Africa		0.039	0.078
	1052.ENR.STOR	Clinical	Africa		0.039	0.078
	1049.THER1.STOR	Clinical	Africa		0.039	0.039

**Notes:**

a USA, United States of America; FLU, fluconazole.

b FLC (fluconazole) MIC data obtained from Chong et al. (2010) and Lai et al. (2016).

c FLB (flubendazole) MICs and MFCs are shown as modes from four biological replicates, each with technical duplicates. A concentration range is given where variable results were obtained.

### Enzo drug library and drug stocks

The Screen-Well FDA-approved drug library was purchased from Enzo Life Sciences (catalogue no. BML-2841; Farmingdale, NY, USA). The 640 compounds in this library were provided at 2 mg/mL in dimethyl sulfoxide (DMSO). Flubendazole (FLB), mebendazole (MEB), benomyl (BEN), nisoldipine (NIS), nifedipine (NIF), felodipine (FEL), niguldipine (NIG), amphotericin B (AMB), itraconazole (ITZ), voriconazole (VRZ) and 5-flucytosine (5FC) were obtained from Sigma-Aldrich (St. Louis, MO, USA). Fluconazole (FLC) was obtained from Cayman Chemical (Ann Arbor, MI, USA). Stock solutions of amphotericin B, itraconazole and voriconazole were prepared at 1.6 mg/mL in water; 5-flucytosine was made at 6.4 mg/mL in water; fluconazole was made at 12.8 mg/mL in water and all remaining drugs were dissolved in DMSO at 2 mg/mL. All compounds were stored at −80 °C.

### Primary drug screening

We evaluated all 640 FDA-approved compounds in the Enzo library for efficacy against *C. deuterogattii* strain R265. In the primary screen, each compound was tested once at 10 μg/mL and once at 40 μg/mL using 96-well flat-bottom microtitre plates. Amphotericin B was included as a standard antifungal control. Untreated cells and broth-only controls were also included. Cell suspensions of *C. deuterogattii* strain R265 were prepared following the reference methods of the Clinical and Laboratory Standards Institute (CLSI) for broth dilution antifungal susceptibility testing of yeasts (Clinical and Laboratory Standards Institute (CLSI), 2008b). Briefly, an isolated colony of *C. deuterogattii* strain R265 from a 2-day old plate was inoculated into 1 mL of Yeast Nitrogen Broth (YNB, pH 7; Sigma-Aldrich, St. Louis, MO, USA). A working cell suspension was prepared at 1 × 10^6^ to 5 × 10^6^ cells/mL by cell counting using a haemocytometer, and was diluted 1:100. Final cell concentrations were 0.5 × 10^3^ to 2.5 × 10^3^ cells/mL with a final volume of 200 μL per well. Plates were incubated at 37 °C (humidified) without agitation for 72 h. Initial ‘hit’ candidates were defined as compounds that visibly inhibited the growth of *C. deuterogattii.*

To identify compounds with the greatest potential for repurposing as novel antifungal agents, compounds that complied with the following were excluded from further assessment: (i) compounds that were active only at concentrations greater than 10 μg/mL; (ii) compounds that were known antineoplastic agents or had otherwise unwanted adverse profiles; and (iii) currently established antifungal agents or non-antifungal agents with antifungal activity that had already been described and assessed at the time of testing.

### Broth microdilution test

The in vitro antifungal activities of ‘hit’ compounds were re-evaluated using the stocks supplied in the library as well as from independently sourced stocks, and testing was extended to additional pathogenic fungal species (Tables 1 and 2). Drug compounds were serially diluted twofold in YNB medium (final concentrations: 0.078125–40 μg/mL). Amphotericin B was included as an antifungal control for reference. Niguldipine, a member of the same class of 1,4-dihydropyridines (calcium channel blocker (CCB)) that had been identified as a non-‘hit’ compound in the screen was included as a negative control for the CCB series. Fungal cell suspensions were prepared following the CLSI standard method for broth dilution antifungal susceptibility testing of yeasts (Clinical and Laboratory Standards Institute (CLSI), 2008b) or filamentous fungi (Clinical and Laboratory Standards Institute (CLSI), 2008a). Plates were incubated without agitation at 35 or 37 °C (humidified) for 48 h for non-*Cryptococcus* species or 72 h for *Cryptococcus* species. The minimum inhibitory concentration (MIC) was determined as the lowest drug concentration at which a compound visibly inhibited growth (100% for FLB, MEB, BEN, NIF, NISO, FELO, NIG, AMB and 5FC; 80% for FLC, ITZ and VRZ). To obtain the minimum fungicidal concentration (MFC), 30 μL from each dilution well at the MIC concentration and higher was spread onto SDA plates. The MFC was defined as the lowest drug concentration that yielded three or fewer colonies after 72 h incubation (Espinel-Ingroff et al., 2002). All microdilution assays were tested in technical duplicates with at least two biological replicates unless otherwise specified.

### Checkerboard microdilution assay

Checkerboard assays determine the interaction between two drugs based on the fractional inhibitory concentration index (FICI) value. Interactions can be synergistic (FICI ≤ 0.5), additive/indifferent (0.5 < FICI ≤ 4) or antagonistic (FICI > 4) (Lai et al., 2016). Briefly, drug A (flubendazole, mebendazole or benomyl) was serially diluted from 0.31 to 0.0024 μg/mL and aliquoted in a 96-well flat-bottom plate and combined with drug B that was serially diluted and aliquoted in the following concentration ranges: amphotericin B (2–0.016 μg/mL); fluconazole (32–0.25 μg/mL); itraconazole (2–0.016 μg/mL); voriconazole (2–0.016 μg/mL); 5-flucytosine (32–0.25 μg/mL).

FICI values were calculated as:

$${\rm{FICI}}\, = \,{{{\rm{MIC\;drug\;A\;in\;combination}}} \over {{\rm{MIC\;drug\;A\;alone}}}} + {{{\rm{MIC\;drug\;B\;in\;combination}}} \over {{\rm{MIC\;drug\;B\;alone}}}}$$

All drug combinations were tested against *C. deuterogattii* strain R265 and *C. neoformans* strain H99 as per CLSI guidelines. All plates were incubated at 37 °C and inhibition was read visually at 72 h.

### Time-kill assay

*Cryptococcus neoformans* strain H99 was grown overnight to exponential phase in YNB media, shaking (180 rpm) at 37 °C. Cells were adjusted to 1 × 10^6^ cells/mL using a haemocytometer and sub-cultured in fresh YNB media. Three hours post sub-culturing, two 50 mL aliquots were taken. One culture was not treated, while the other was treated with 0.06 μg/mL flubendazole (1.5 × MIC). Cultures were returned to the shaking incubator and monitored for cell growth at 2, 3, 4, 5, 6, 9 and 24 h post-treatment. At each time point, each culture was sampled in technical duplicates, serially diluted in milli-Q water and spread onto SDA plates. All plates were incubated at 37 °C and observed at 48 h and 72 h for viability counts.

## Results

### Primary drug screening identifies non-antifungal compounds with anti-cryptococcal activity

**Figure 1:**
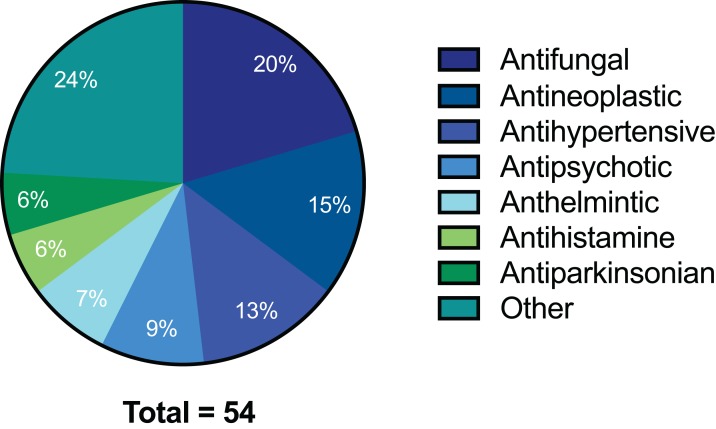
Pie chart of drug compound classes that inhibited the growth of *C. deuterogattii* strain R265 at 10 μg/mL. A total of 54 compounds inhibited the growth of *C. deuterogattii* strain R265 at 10 μg/mL. Classes of drugs included: antifungal (20%); antineoplastic (15%); antihypertensive (13%); antipsychotic (9%); anthelmintic (7%); antihistamine (6%); antiparkinsonian (6%); other (24%).

**Figure 2:**
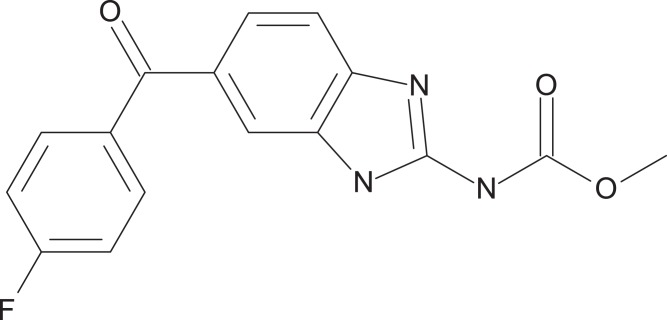
Chemical structure of flubendazole. Flubendazole was drawn with BIOVIA Draw 2017 R2 (Version 17.2; Dassault Systèmes, San Diego, CA, USA).

**Figure 3:**
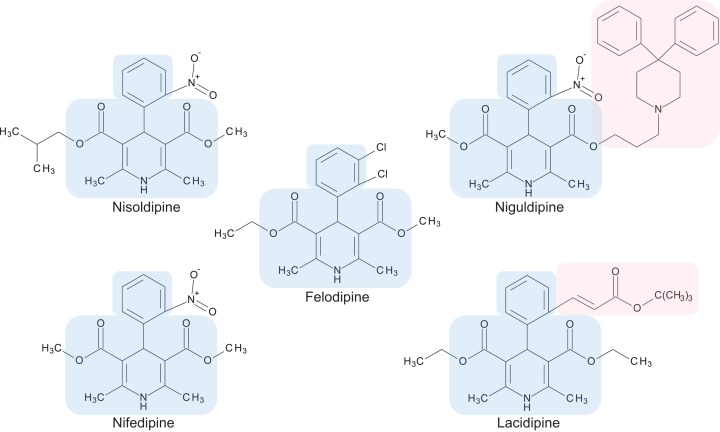
Chemical structures of 1,4-dihydropyridine calcium channel blockers: nisoldipine, nifedipine, felodipine, niguldipine and lacidipine. The core structure shared by members of the 1,4-dihydropyridine class is shaded in blue. Large side groups are shaded in pink. Chemical structures were drawn with BIOVIA Draw 2017 R2 (Version 17.2; Dassault Systèmes, San Diego, CA, USA).

The preliminary screening of the Enzo library revealed a diverse variety of compounds that inhibited the growth of *C. deuterogattii* strain R265 (Fig. 1; Fig. S1; Table S1). A significant proportion (15%) of ‘hits’ from this initial screen were antineoplastic drugs. This antifungal affect may be attributed to the cytotoxic nature of these compounds since cryptococcal and humans cells are eukaryotic and may share cellular structures and processes that result in non-selective toxicity. For this reason, this group of drugs were not pursued as potential antifungal candidates. Using the selection criteria described in the “Methods” section, 16 of the remaining compounds identified in the primary screen were short-listed for further evaluation by MIC assays against *C. deuterogattii*. Antifungal activity was confirmed for ten compounds using drug stocks sourced from the library. The most potent compounds (MICs < 5 μg/mL) were aripiprazole, nisoldipine, oxatomide, flubendazole, nifedipine and anethole-trithione. Lack of commercial availability restricted further evaluation to flubendazole, an anthelmintic drug (Fig. 2), and nisoldipine and nifedipine, which are antihypertensive CCBs (Fig. 3). Felodipine and niguldipine, which are CCBs structurally related to nifedipine and nisoldipine, were also included in the assay.

### In vitro spectrum of antifungal activity of CCBs and flubendazole

To investigate the spectrum of antifungal activity, MICs were determined for nisoldipine, nifedipine, felodipine, niguldipine and flubendazole against nine additional strains from four fungal species (Table 1). MIC assays using independently sourced drug stocks confirmed the MICs established for *C. deuterogattii* strain R265 and demonstrated a diverse range of antifungal activities against other fungal strains and species (Table 1). Flubendazole was the most potent compound against *Cryptococcus* species with MICs as low as 0.078 μg/mL, but was ineffective even at the highest tested concentration (40 μg/mL) against other fungal species. The series of CCBs presented an interesting array of activities across the fungal species. Nifedipine demonstrated consistent antifungal activity against the *Cryptococcus* species (5–10 μg/mL) and inhibited *Candida glabrata*, an emerging fungal pathogen, with an MIC of 20 μg/mL. In contrast, nisoldipine had higher and more variable MICs against different *Cryptococcus* species (5–40 μg/mL) and was the only drug that had activity against all nine fungal species. Niguldipine was not effective at the highest tested concentration against the fungal strains in this assay, while felodipine demonstrated activity against the *Cryptococcus* species with MICs between those of nifedipine and nisoldipine, but was only active against *Cryptococcus* species.

### Antifungal activities of flubendazole

To test whether flubendazole is broadly active across *Cryptococcus* species, we extended the study to encompass all members of the *C. gattii* species complex, as well as the major molecular genotypes of *C. neoformans* (Table 2; Table S2), including veterinary, clinical and environmental isolates from various geographic locations. Flubendazole inhibited all isolates of both *Cryptococcus* species at consistently low concentrations (MIC 0.039–0.156 μg/mL). Furthermore, for isolates where fluconazole MIC data were available, flubendazole was found to be equally effective regardless of fluconazole susceptibility (Table 2; Table S2).

**Figure 4:**
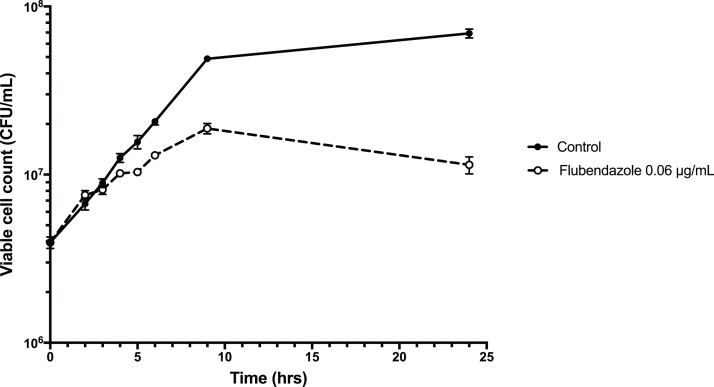
Time-kill assay of *C. neoformans* strain H99 treated with flubendazole. *C. neoformans* cells in logarithmic growth phase were treated with 0.06 μg/mL flubendazole and incubated at 37 °C with shaking. The growth of treated cells tracked the untreated control cells for 3–4 h post-treatment and began to slow thereafter. Viability declined markedly by 9 h and did not recover by 24 h. Growth curve was generated using Prism 7 (Version 7.0b; GraphPad Software, La Jolla, CA, USA).

To explore the inhibitory effect of flubendazole on *Cryptococcus* cells, cell viability was assessed using a time-kill assay and determining the MFC in a microwell format. Analysis by time-kill curve over 24 h demonstrated that *Cryptococcus* cells exposed to flubendazole had slowly declining growth for up to 9 h post-treatment prior to loss of viability (Fig. 4; Table S3). Microbroth dilution assays generated MFC values that were similar to the respective MIC values (MFC 0.039–0.156 μg/mL; Table 2).

To explore interactions with current, commonly used antifungals, flubendazole and the other benzimidazole agents mebendazole and benomyl was tested in combination with amphotericin B, fluconazole, itraconazole, voriconazole and 5-flucytosine against *C. deuterogattii* and *C. neoformans* in a checkerboard assay. All combinations produced additive/indifferent results and no synergistic or antagonistic interactions were seen (Table 3; Tables S4–S5).

**Table 3:** Fractional inhibitory concentration index values of flubendazole, mebendazole and benomyl in combination with various antifungals against *C. neoformans* H99 and *C. deuterogattii* R265.

*Cryptococcus* species	Benzimidazole	Fractional inhibitory concentration index^a^				
		AMB	FLC	ITZ	VRZ	5FC
***C. neoformans* H99**	**Flubendazole**	(0.75–2)	(1.5–2)	(1.5–3)	(1–1.25)	2
	**Mebendazole**	(1–2)	1.5	(1–2.5)	(1–1.125)	(1.5–2)
	**Benomyl**	(0.75–1.5)	(1.25–1.5)	(1.25–1.5)	(0.75–1.125)	(1.25–1.5)
***C. deuterogattii* R265**	**Flubendazole**	(1.5–3)	(2–3)	(1.5–2.5)	(1–1.5)	(1–1.5)
	**Mebendazole**	(1.5–2.5)	(1.5–2.5)	(1–2.5)	(0.75–1.5)	1.5
	**Benomyl**	(1–2.25)	(1.25–1.5)	(1.25–1.5)	(0.625–1)	(1–1.25)

**Note:**

a AMB, amphotericin B; FLC, fluconazole; ITZ, itraconazole; VRZ, voriconazole; 5FC, 5-flucytosine. Data shown were produced from two independent replicates.

## Discussion

Despite the major health problems caused by cryptococcal infections, there is a lack of new antifungal drugs. Promising, albeit limited, research has demonstrated the potential for repurposing drugs and the implications of this approach for treating cryptococcal infections have been highlighted (Butts et al., 2013; Dehdashti et al., 2013; Joffe et al., 2017; Rabjohns et al., 2013; Rhein et al., 2016; Samantaray et al., 2016; Spitzer et al., 2011). Most studies have screened the Prestwick Chemical Library (*n* = 1,120), the Library of Pharmaceutically Active Compounds (*n* = 1,280) and the National Institutes of Health clinical collection (*n* = 727). Within the last decade, these drug screening studies have led to the discovery of multiple non-antifungal compounds with antifungal activities, which were often novel and fungicidal (Butts et al., 2013; Dehdashti et al., 2013; Joffe et al., 2017; Rabjohns et al., 2013; Samantaray et al., 2016; Spitzer et al., 2011). Some have demonstrated the ability to target intracellular *C. neoformans* in macrophages and cryptococcal biofilms and to act synergistically with the widely-used drug fluconazole (Butts et al., 2013; Dehdashti et al., 2013; Joffe et al., 2017; Rabjohns et al., 2013; Samantaray et al., 2016; Spitzer et al., 2011). However, these studies have focused on *C. neoformans* with only one reporting on *C. deuterogattii* strain R265 (Spitzer et al., 2011). There can be significant differences in target specificity among closely related genera and species of pathogenic yeasts (Chong et al., 2010; Spitzer et al., 2011). Thus, while *C. neoformans* and *C. deuterogattii* are sibling species, drugs may work differently between them. Here we report the first screening of a 640-compound FDA-approved library against *C. deuterogattii* strain R265, with subsequent testing of additional pathogenic cryptococci (encompassing an extensive list of *C. neoformans* species and the entire *C. gattii* species complex) and other fungi.

### Repurposing CCBs as antifungal agents

Calcium channel blockers, widely used in the treatment of hypertension, are broadly divided into dihydropyridines and non-dihydropyridines classes (Catterall, 2011). Our screen identified nifedipine, felodipine and nisoldipine (Table 1; Fig. 3) as having antifungal activity against *Cryptococcus*. These are all 1,4-dihydropyridines and therefore present as intriguing leads for a potential new class of antifungal drugs.

Members of the 1,4-dihydropyridine CCB group share a core phenyl group attached to a 1,4-dihydropyridine that has two ester side groups (Fig. 3). Variations in side groups may account for the diverse antifungal activities observed, as compounds with the bulkier side groups (lacidipine and niguldipine) were ineffective against *C. deuterogattii*. Nifedipine, felodipine, nisoldipine and niguldipine target L-type calcium channels—one of five types of voltage-gated calcium channels (VGCCs) designated L-, N-, P/Q-, R- and T-type (Catterall, 2011). In the model yeast *Saccharomyces cerevisiae*, Cch1 and Mid1 are homologs of the mammalian VGCCs, and L-type CCBs are known to inhibit fungal growth via a Cch1–Mid1 protein channel complex located in the plasma membrane (Prole & Taylor, 2012; Teng et al., 2008). Cch1 acts as the calcium channel and has homologs in almost all fungi, while Mid1 is a regulatory subunit that interacts with Cch1 to mediate calcium influx (Hong et al., 2013). Hence, it appears likely that the antifungal effects of CCBs involve the Cch1–Mid1 calcium channel.

Our findings support a growing body of evidence suggesting a crucial role of calcium regulation and calcium channels in determining the growth and survival of yeasts under stressful conditions (Hong et al., 2010; Liu et al., 2006; Peiter et al., 2005). In *C. neoformans*, Cch1 facilitates calcium entry with Mid1 when there is a depletion of intracellular calcium (Hong et al., 2010). In the presence of the calcium chelator BAPTA, the growth of *Δcch1* mutants is markedly diminished at elevated temperature (25–38.5 °C), a key virulence factor that is critical for human infection (Liu et al., 2006). Cch1 and Mid1 also appear linked to the regulation of intracellular redox under cell stress, as *S. cerevisiae Δcch1* and *Δmid1* (single- and double-knockout) mutants are sensitive to cold stress (10 °C) and iron toxicity due to a compromised ability to cope with oxidative stress (Peiter et al., 2005). Cell growth can be re-established following supplementation with either 10 mM calcium or reduced glutathione (an antioxidant), indicating that a functional Cch1–Mid1 complex is essential for stress tolerance (Peiter et al., 2005).

As well as possessing antifungal properties, CCBs have also been shown to act synergistically with fluconazole against fluconazole-resistant and -susceptible strains of *C. albicans* (Liu et al., 2016). Interestingly, CCBs alone or in combination with fluconazole do not significantly alter the gene expression level of *CCH1* and *MID1* (Liu et al., 2016) suggesting that either the cell is not sensing a disruption to calcium homeostasis, or CCBs act on targets other than calcium channels. Recently, a screen of the Prestwick Chemical Library® of FDA-approved small molecules identified fendiline hydrochloride, a non-dihydropyridine CCB, as having unique activity to potentiate phagosomal killing of intracellular *C. neoformans* in host macrophages (Samantaray et al., 2016). Although it has a very different structure to the antifungal CCBs identified here, fendiline is an L-type CCB, suggesting this class of CCBs may be the most promising for antifungal drug development. Furthermore, the antifungal activity of L-type CCBs in combination with standard antifungals have been characterised in *C. albicans* and *Aspergillus* (Afeltra et al., 2004; Liu et al., 2016). As hypertensive agents, L-type CCBs are currently administered in long term use with relatively minimal side effects (Chrysant, 2003). Of clinical relevance, some CCBs including nifedipine and nisoldipine can traverse the blood–brain barrier and may therefore be able to treat cryptococcal meningitis (Gaggi, Cont & Gianni, 1993). We therefore suggest L-type CCBs are promising leads for repurposing and redevelopment as potential new antifungal agents.

### Repurposing flubendazole as an anti-cryptococcal agent

Flubendazole is an anthelmintic drug belonging to a family of benzimidazole carbamates. First introduced in 1980, it is currently used to treat parasitic gastrointestinal worm infections in humans and animals (Fig. 2) (Spagnuolo et al., 2010). Flubendazole acts by binding to and inhibiting the polymerisation of tubulin, a cytoskeletal protein that polymerises to form microtubules (Chatterji et al., 2011; Cruz & Edlind, 1997; Spagnuolo et al., 2010). Microtubules play major roles in various cell processes including division, motility, adhesion and intracellular transport (Chatterji et al., 2011). Benzimidazoles have been found to act selectively against fungal tubulin and not mammalian tubulin, possibly due to differences in their β-tubulin sequence (Cruz & Edlind, 1997).

Benzimidazoles were originally developed as agricultural fungicides prior to their use as anthelmintic drugs (Horton, 2000). While the antifungal activities of benzimidazoles and their potential to be developed as anti-cryptococcal agents have been known for over two decades (Patent US5434163) (Cruz, Bartlett & Edlind, 1994; Del Poeta et al., 1998; Edlind & Cruz, 1995), no further development as antifungal agents has occurred since 1998 (Del Poeta et al., 1998). Our study indicated that flubendazole is fungicidal at extremely low concentrations against *C. neoformans* and *C. deuterogattii* (Table 2). Our findings show that flubendazole equally inhibits pathogenic and environmental isolates of *Cryptococcus*, as well as strains that are resistant to fluconazole. This indicates that flubendazole may be an effective treatment against genetically and physiologically diverse *Cryptococcus* species and strains. As resistance to fluconazole is frequently induced during prolonged treatment and has been associated with clinical failure in *Cryptococcus*-infected patients (Bicanic et al., 2006; Jarvis et al., 2014; Smith et al., 2015), flubendazole may have promise as a second-line therapy when fluconazole fails, particularly in resource-poor regions where there are often no suitable alternatives.

Flubendazole interferes with normal cell growth as early as 3 h post-treatment and continues to render treated *Cryptococcus* cells unviable (Fig. 4), and in a microwell format with 72 h incubation the MFC values were either the same or one increment higher than the respective MIC values (Table 2). This indicates that flubendazole is a fungicidal drug, and with the correct clinical administration, it could effectively clear fungal burden. As high fungal burden is a strong determinant for mortality and overall poor clinical outcomes, and currently amphotericin B is the only fungicidal agent that is effective against cryptococcosis, this reinforces the potential utility of flubendazole as an anti-cryptococcal agent (Jarvis et al., 2014). It is noted however, that unlike amphotericin B, flubendazole does not act synergistically with other standard antifungal agents, consistent with previously published data (Table 3) (Joffe et al., 2017).

The current findings support a recent screening of 727 compounds in the National Institute of Health Clinical Collection (Joffe et al., 2017). Flubendazole and three related anti-helminthic benzimidazoles (mebendazole, albendazole and triclabendazole; sharing the benzimidazole scaffold) were among 17 compounds active against *C. neoformans* (Joffe et al., 2017). However, that study focused on mebendazole on the basis that the drug is able to penetrate the blood–brain barrier. Low MIC and MFC values were reported, and efficacy against biofilms and against cryptococcal cells internalised in macrophages were shown, along with additive activity when combined with amphotericin B (Joffe et al., 2017). Flubendazole may mirror the antifungal capabilities observed for mebendazole as they share structural similarities, and there is evidence that like mebendazole, flubendazole can traverse the blood–brain barrier (Téllez-Girón et al., 1984) making these compounds favourable candidates for the treatment of cryptococcal meningitis. However, as flubendazole is formulated to treat gastrointestinal worms, it is not yet known whether it would be able to reach therapeutic concentrations in the brain required to elicit an antifungal effect.

The precise mechanism underlying the antifungal activity of flubendazole is unclear, however it is thought that it targets β-tubulin in *C. neoformans* (Cruz & Edlind, 1997). The kill-curve demonstrated initial growth after treatment followed by declining viability and death at 9 h, which is consistent with a mode of action that works by interfering with cell growth and division. In *Echinococcus granulosus*, a tapeworm parasite, flubendazole has been found to disrupt a wide spectrum of cellular homeostatic mechanisms, including energy metabolism and calcium homeostasis. In the latter it appears to increase cytosolic calcium from extracellular and intracellular stores (Cumino, Elissondo & Denegri, 2009), demonstrating an interesting overlap with the CCBs outlined above.

Overall, flubendazole (and more generally the benzimidazole scaffold) may be a promising starting point for the treatment of cryptococcal disease. This view is further encouraged by a lack of evidence of serious adverse side effects in patients undergoing anti-helminthic treatment and in mice experimentally treated with flubendazole (Ceballos et al., 2009; Feldmeier et al., 1982; Lassègue et al., 1984; Téllez-Girón et al., 1984). In preliminary cryptococcal meningitis studies, flubendazole demonstrated fungicidal activity in a hollow fibre infection model and efficacy in vivo, reducing fungal burden in both a rabbit and mice model (Nixon et al., 2018). However, whether flubendazole can accumulate in the brain to concentrations that are clinically achievable and able to elicit an antifungal effect, and whether this can be sustained in the absence of adverse reactions, remains unknown and requires more extensive in vivo testing.

## Conclusion and Perspectives

This study, and others like it, demonstrate the utility of drug repurposing to discover novel agents for the treatment of neglected diseases like cryptococcosis. L-type CCBs and anti-helminthic benzimidazoles have emerged in different drug screens and appear particularly promising as both are registered for use in other applications, are well tolerated and appear able to traverse the blood–brain barrier. Phenotypic screening approaches have repeatedly demonstrated utility, ensuring that identified drug candidates possess the desired phenotype of inhibiting growth of the pathogen prior to further mechanistic investigations. In addition to serendipitously finding molecules or compounds that can be used directly in the clinic, repurposing strategies may identify related classes of compounds with activity and thus provide a scaffold for subsequent medicinal chemistry to initiate lead drug discovery programs.

Currently, it costs billions of dollars for a drug to successfully obtain FDA-approval and reach market. To help alleviate this financial risk and provide an incentive, there are moves by regulatory agencies in the United States and Europe to encourage academic and pharmaceutical research to bring new antimicrobials to market. For example, the GAIN Act (Generating Antibiotic Incentives Now) grants companies manufacturing a ‘qualified infectious disease product’ an additional period of market exclusivity, with or without a patent, to ensure a better return on their investment. This applies to the repurposing of existing drugs with new indications, and should encourage pharmaceutical companies to develop some of these newly identified agents as therapeutics for fungal diseases, which are urgently required in global health.

## Supplemental Information

10.7717/peerj.4761/supp-1 Supplemental Information 1 Pie chart of all drug compound classes that inhibited the growth of *C. deuterogattii* strain R265 at 10 μg/mL and or at 40 μg/mL. A total of 109 compounds inhibited the growth of *C. deuterogattii* strain R265 at 10 μg/mL and or 40 μ g/mL. Classes of drugs included: antineoplastic (15%); antifungal (13%); antihypertensive (10%); antipsychotic (8%); antihistamine (7%); antidepressant (7%); anthelmintic (6%); antiparkinsonian (5%); other (32%).

10.7717/peerj.4761/supp-2 Supplemental Information 2 List of 109 hit compounds identified from the primary screen on the Enzo library against * C. deuterogattii* R265.

10.7717/peerj.4761/supp-3 Supplemental Information 3 Minimum Inhibitory Concentration and Minimum Fungicidal Concentration of flubendazole across various strains and species of *Cryptococcus*. MIC, minimum inhibitory concentration; MFC, minimum fungicidal concentration.

10.7717/peerj.4761/supp-4 Supplemental Information 4 Viability counts of *Cryptococcus neoformans* H99 treated with flubendazole (0.06 μg/mL) over 24 h. Raw data from four independent replicates.

10.7717/peerj.4761/supp-5 Supplemental Information 5 Minimum Inhibitory Concentrations of flubendazole, mebendazole and benomyl alone and in combination with known antifungal agents amphotericin B, fluconazole, itraconazole, voriconazole and 5-flucytosine, against *C. neoformans* H99. Raw data from two independent replicates. Drug A are antifungal agents (AMB, amphotericin B; FLC, fluconazole; ITZ, itraconazole; VOR, voriconazole; 5FC, 5-flucytosine) and Drug B are benzimidazole agents (FLB, flubendazole; MEB, mebendazole; BEN, benomyl).
^a^ MIC of Drug A or B alone
^b^ MIC of Drug A in combination with a benzimidazole
^c^ MIC of Drug B with an antifungal agent

10.7717/peerj.4761/supp-6 Supplemental Information 6 Minimum Inhibitory Concentrations of flubendazole, mebendazole and benomyl alone and in combination with knownantifungal agents amphotericin B, fluconazole, itraconazole, voriconazole and5-flucytosine, against *C. deuterogattii* R265. Raw data shows the results from two independent replicates. Drug A are antifungal agents (AMB, amphotericin B; FLC, fluconazole; ITZ, itraconazole; VOR, voriconazole; 5FC, 5-flucytosine) and Drug B are benzimidazole agents (FLB, flubendazole; MEB, mebendazole; BEN, benomyl).
^a^ MIC of Drug A or B alone
^b^ MIC of Drug A in combination with a benzimidazole
^c^ MIC of Drug B with an antifungal agent

## Additional Information and Declarations

### Data Availability

The following information was supplied regarding data availability: The raw data have been uploaded as a Supplemental File.
