# Supplementary material for: Repurposing drugs to fast-track therapeutic agents for the treatment of cryptococcosis
Source: PeerJ. 2018 May 4;6:e4761. doi: 10.7717/peerj.4761 (PMC5937474; doi:10.7717/peerj.4761)
Supplement: Supplemental Information 1 — A total of 109 compounds inhibited the growth of C. deuterogattii strain R265 at 10 μg/mL and or 40 μ g/mL. Classes of drugs included: antineoplastic (15%); antifungal (13%); antihypertensive (10%); antipsychotic (8%); antihistamine (7%); antidepressant (7%); anthelmintic (6%); antiparkinsonian (5%); other (32%). [file peerj-06-4761-s001.pdf]

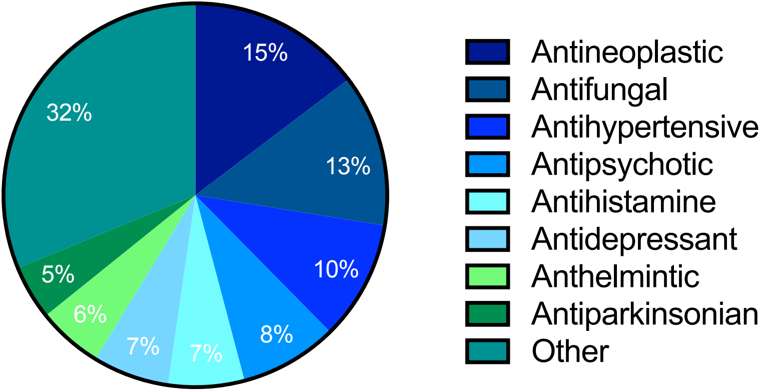
15%
13%
10%
8%
7%
7%
6%
5%
32%
Antineoplastic
Antifungal
Antihypertensive
Antipsychotic
Antihistamine
Antidepressant
Anthelmintic
Antiparkinsonian
Other

Total = 109
