# Supplementary material for: Repurposing drugs to fast-track therapeutic agents for the treatment of cryptococcosis
Source: PeerJ. 2018 May 4;6:e4761. doi: 10.7717/peerj.4761 (PMC5937474; doi:10.7717/peerj.4761)
Supplement: Supplemental Information 2 [file peerj-06-4761-s002.pdf]

No.	Drug	Active concentration (μg/mL)		Therapeutic classification
**1**	Fluspirilene		10	Antipsychotic
**2**	Aripiprazole		10	Antipsychotic
**3**	Fluphenazine	40	10	Antipsychotic
**4**	Haloperidol	40		Antipsychotic
**5**	Butaclamol (+)	40		Antipsychotic
**6**	Trifluperidol	40		Antipsychotic
**7**	Trifluoperazine·2HCl	40	10	Antipsychotic
**8**	Thioridazine	40	10	Antipsychotic
**9**	Clothiapine	40		Antipsychotic
**10**	Nisoldipine	40	10	Antihypertensive
**11**	Lacidipine		10	Antihypertensive
**12**	Methyldopa	40	10	Antihypertensive
**13**	Naftopidil·2HCl	40		Antihypertensive
**14**	Felodipine	40	10	Antihypertensive
**15**	Nifedipine	40	10	Antihypertensive
**16**	Nitrendipine	40		Antihypertensive
**17**	Doxazosin	40	10	Antihypertensive
**18**	Olmesartan	40	10	Antihypertensive
**19**	Trequinsin·HCl	40		Antihypertensive
**20**	Fenoldopam	40		Antihypertensive
**21**	Flubendazole		10	Anthelmintic
**22**	Albendazole		10	Anthelmintic
**23**	Oxfendazole	40	10	Anthelmintic
**24**	Ricobendazole	40		Anthelmintic
**25**	Quinacrine	40	10	Anthelmintic
**26**	Fenbendazole	40		Anthelmintic
**27**	Clemastine	40	10	Antihistamine
**28**	Terfenadine	40	10	Antihistamine
**29**	Promethazine	40		Antihistamine
**30**	Loratadine	40		Antihistamine
**31**	Cyproheptadine	40		Antihistamine
**32**	Astemizole	40		Antihistamine
**33**	Oxatomide	40	10	Antihistamine
**34**	Amorolfine	40	10	Antifungal
**35**	Butenafine	40	10	Antifungal
**36**	Sertaconazole		10	Antifungal
**37**	Terbinafine	40	10	Antifungal
**38**	Nystatin	40		Antifungal
**39**	Oxiconazole		10	Antifungal
**40**	Myclobutanil	40	10	Antifungal
**41**	Miconazole		10	Antifungal
**42**	Fluconazole	40		Antifungal
**43**	Bifonazole		10	Antifungal
**44**	Climbazole	40	10	Antifungal

**45**	Butoconazole nitrate	40	10	Antifungal
**46**	Tioconazole	40		Antifungal
**47**	Ketoconazole	40	10	Antifungal
**48**	Carbidopa	40		Antiparkinsonian
**49**	Levodopa	40	10	Antiparkinsonian
**50**	Entacapone	40		Antiparkinsonian
**51**	Benserazide	40	10	Antiparkinsonian
**52**	Apomorphine R (-)	40	10	Antiparkinsonian
**53**	Imipramine	40		Antidepressant
**54**	Amoxapine	40		Antidepressant
**55**	Mianserin·HCl	40		Antidepressant
**56**	Nefazodone	40		Antidepressant
**57**	Paroxetine	40		Antidepressant
**58**	Fluoxetine	40		Antidepressant
**59**	Maprotiline·HCl	40		Antidepressant
**60**	Vincristine	40	10	Antineoplastic
**61**	Toremifene	40	10	Antineoplastic
**62**	Vindesine sulfate	40	10	Antineoplastic
**63**	Docetaxil	40	10	Antineoplastic
**64**	Vinorelbine	40		Antineoplastic
**65**	Lomustine	40		Antineoplastic
**66**	Camptothecin	40		Antineoplastic
**67**	5-Fluorouracil	40	10	Antineoplastic
**68**	Idarubicin	40		Antineoplastic
**69**	Aclarubicin	40		Antineoplastic
**70**	Mitoxantrone	40		Antineoplastic
**71**	Tamoxifen	40	10	Antineoplastic
**72**	Bleomycin	40	10	Antineoplastic
**73**	Miltefosine	40	10	Antineoplastic
**74**	Sodium Phenylbutyrate	40		Antineoplastic
**75**	Vinblastine	40		Antineoplastic
**76**	Auranofin	40	10	Antiinflammatory
**77**	Docebenone	40	10	Antiinflammatory
**78**	Capsaicin	40		Analgesic
**79**	Simvastatin	40		Antihyperlipidemic
**80**	Cerivastatin	40	10	Antihyperlipidemic
**81**	Lorglumide	40		Antiulcerative
**82**	Omeprazole	40		Antiulcerative
**83**	Tacrine	40		Nootropic
**84**	Idebenone	40		Nootropic
**85**	Melengestrol Acetate	40		Progestogen
**86**	Progesterone	40		Progestogen
**87**	Levallorphan	40	10	Opioid antagonist
**88**	Naltriben methanesulfonate hydrate	40	10	Opioid antagonist

**89**	Celecoxib	40		Analgesic
**90**	Fumagillin	40		Antiprotozoal
**91**	Pentamidine	40		Antiprotozoal
**92**	Anethole-trithione	40	10	Choleretic
**93**	Ticlopidine	40	10	Antithrombotic
**94**	Efavirenz	40	10	Antiviral
**95**	Epinephrine-(+)- L (-)	40		Bronchodilator, cardiostimulator
**96**	Norepinephrine-(+)-tartrate L (-)	40		Antihypotensive
**97**	Vitamin A acetate	40	10	Vitamin
**98**	Riluzole·HCl	40		Neuroprotective
**99**	Harmine	40		CNS stimulant
**100**	Flutamide	40		Antiandrogen
**101**	Clomiphene	40	10	Gonad stimulant
**102**	Flunarizine·2HCl		10	Vasodilator
**103**	Artemisinin	40	10	Antimalarial
**104**	Disulfiram	40	10	Alcohol deterrent
**105**	Leflunomide	40		Antirheumatic
**106**	Prothionamide	40		Antitubercular
**107**	Chlorpromazine	40		Antiemetic
**108**	Lomofungin	40		Antibiotic
**109**	Bepridil·HCl	40		Antianginal
