# Supplementary material for: Repurposing drugs to fast-track therapeutic agents for the treatment of cryptococcosis
Source: PeerJ. 2018 May 4;6:e4761. doi: 10.7717/peerj.4761 (PMC5937474; doi:10.7717/peerj.4761)
Supplement: Supplemental Information 3 — MIC, minimum inhibitory concentration; MFC, minimum fungicidal concentration. [file peerj-06-4761-s003.pdf]

Species	Molecular genotype	Strain	MIC (ug/mL)					MFC (ug/mL)				
			Rep - 1	*Rep - 2*	*Rep - 3*	*Rep - 4*	Mode	*Rep - 1*	*Rep - 2*	*Rep - 3*	*Rep - 4*	Mode
C. gattii	VGI	ENV316	0.078125	0.078125	0.078125	-	0.078	0.1563	0.1563	0.078125	-	0.156
		PNG14	0.078125	0.039063	0.078125	-	0.078	0.078125	0.078125	0.078125	-	0.078
		2005/215	0.078125	0.078125	0.078125	-	0.078	0.078125	0.078125	0.078125	-	0.078
		V15/571_103	0.078125	0.078125	0.078125	-	0.078	0.078125	0.15625	0.078125	-	0.078
C. deuterogattii	VGII	97/170	0.078125	0.078125	0.078125	-	0.078	0.078125	0.078125	0.078125	-	0.078
		R265	0.078125	0.078125	0.078125	-	0.078	0.078125	0.078125	0.078125	-	0.078
		CBS1930	0.039063	0.078125	0.078125	-	0.078	0.078125	0.078125	0.078125	-	0.078
		ICB184	0.039063	0.039063	0.039063	-	0.039	0.039063	0.039063	0.039063	-	0.039
		14.1431	0.039063	0.019531	0.039063	-	0.039	0.078125	0.039063	0.078125	-	0.078
		LA499	0.039063	0.019531	0.039063	-	0.039	0.039063	0.039063	0.039063	-	0.039
		V5	0.039063	0.039063	0.078125	0.019531	0.039	0.078125	0.039063	0.078125	0.039063	0.078
		03-201073	0.078125	0.039063	0.078125	0.039063	0.078	0.078125	0.078125	0.078125	0.078125	0.078
C. bacillisporus	VGIII	VBP62270	0.039063	0.078125	0.078125	-	0.078	0.078125	0.078125	0.078125	-	0.078
		97/427	0.15625	0.15625	0.15625	-	0.156	0.01563	0.3125	0.3125	-	0.313
		WM161	0.078125	0.15625	0.15625	-	0.156	0.0781	0.15625	0.15625	-	0.156
		B13C	0.078125	0.15625	0.078125	-	0.078	0.15625	0.15625	0.078125	-	0.16
C. tetragattii	VGIV	MMRL3013	0.078125	0.15625	0.039063	-	-	0.078125	0.15625	0.039063	-	-
		M250	0.15625	0.3125	0.15625	-	0.156	0.15625	0.3125	0.15625	-	0.16
		WM779	0.039063	0.078125	0.078125	-	0.078	0.078125	0.15625	0.078125	-	0.08
		MMRL2650	0.039063	0.039063	0.039063	-	0.039	0.078125	0.078125	0.078125	-	0.078
C. neoformans	VNI	1043.ENR.STOR	0.078125	0.078125	0.078125	-	0.078	0.15625	0.078125	0.15625	-	0.156
		1020.CLIN1	0.078125	0.039063	0.039063	-	0.039	0.078125	0.078125	0.078125	-	0.078
		H99	0.039063	0.019531	0.039063	0.019531	0.039	0.039063	0.039063	0.039063	0.019531	0.039
		WM625	0.019531	0.019531	0.039063	0.019531	0.020	0.078125	0.039063	0.039063	0.039063	0.039
		WM385	0.039063	0.039063	0.078125	0.039063	0.039	0.039063	0.019531	0.039063	0.039063	0.039
C. neoformans	VNII	1023.ENR	0.039063	0.039063	0.039063	-	0.039	0.078125	0.078125	0.078125	-	0.078
		1045.ENR.STOR	0.078125	0.078125	0.039063	-	0.078	0.15625	0.15625	0.15625	-	0.156
C. neoformans	VNBI	1050.ENR.CLIN	0.078125	0.15625	0.078125	-	0.078	0.15625	0.15625	0.15625	-	0.156
C. neoformans	VNBII	1033.ENR	0.039063	0.039063	0.039063	-	0.039	0.078125	0.078125	0.078125	-	0.078
		1052.ENR.STOR	0.039063	0.039063	0.039063	-	0.039	0.078125	0.078125	0.039063	-	0.078

		1049.THER1.STOR	0.039063	0.039063	0.039063	-	0.039	0.039063	0.078125	0.039063	-	0.039
