# Supplementary material for: Repurposing drugs to fast-track therapeutic agents for the treatment of cryptococcosis
Source: PeerJ. 2018 May 4;6:e4761. doi: 10.7717/peerj.4761 (PMC5937474; doi:10.7717/peerj.4761)
Supplement: Supplemental Information 4 — Raw data from four independent replicates. [file peerj-06-4761-s004.pdf]

Time (hr)	Control (CFU/mL)				Flubendazole 0.06 µg/mL (CFU/mL)			
0	4.45E+06	3.05E+06	4.25E+06	4.05E+06	4.45E+06	3.05E+06	4.25E+06	4.05E+06
2	5.40E+06	6.95E+06	8.05E+06	6.45E+06	7.35E+06	6.40E+06	8.65E+06	7.85E+06
3	8.65E+06	7.60E+06	1.01E+07	9.30E+06	7.00E+06	9.30E+06	7.65E+06	8.60E+06
4	1.30E+07	1.10E+07	1.45E+07	1.18E+07	1.08E+07	9.90E+06	1.04E+07	9.60E+06
5	1.33E+07	1.41E+07	1.97E+07	1.55E+07	1.02E+07	9.75E+06	1.14E+07	1.01E+07
6	2.18E+07	1.79E+07	2.12E+07	2.18E+07	1.19E+07	1.32E+07	1.35E+07	1.35E+07
9	4.70E+07	4.75E+07	5.35E+07	4.75E+07	1.61E+07	2.21E+07	1.96E+07	1.74E+07
24	6.25E+07	6.45E+07	8.10E+07	6.85E+07	1.03E+07	1.42E+07	1.29E+07	8.30E+06
