# Supplementary material for: Repurposing drugs to fast-track therapeutic agents for the treatment of cryptococcosis
Source: PeerJ. 2018 May 4;6:e4761. doi: 10.7717/peerj.4761 (PMC5937474; doi:10.7717/peerj.4761)
Supplement: Supplemental Information 6 — Raw data shows the results from two independent replicates. Drug A are antifungal agents (AMB, amphotericin B; FLC, fluconazole; ITZ, itraconazole; VOR, voriconazole; 5FC, 5-flucytosine) and Drug B are benzimidazole agents (FLB, flubendazole; MEB, mebendazole; BEN, benomyl). a MIC of Drug A or B alone b MIC of Drug A in combination with a benzimidazole c MIC of Drug B with an antifungal agent [file peerj-06-4761-s006.pdf]

C. deuterogattii R265 – replicate 1

Drug A	MIC alone (µg/mL) [a]	MIC combo (µg/mL) [b]				
		FLB	MEB	BEN		
AMB	1	0.5	0.5	0.5		
FLC	8	8	4	8		
ITZ	2	1	1	1		
VOR	0.5	0.25	0.25	0.25		
5FC	16	8	8	8		
**Drug B**	**MIC alone (µg/mL) [a]**	**MIC combo (µg/mL) [c]**				
		**AMB**	**FLC**	**ITZ**	**VOR**	**5FC**
FLB	0.078125	0.078125	0.15625	0.15625	0.078125	0.078125
MEB	0.078125	0.078125	0.15625	0.15625	0.078125	0.078125
BEN	10	5	5	10	5	5

C. deuterogattii R265 – replicate 2

Drug A	MIC alone (µg/mL) [a]	MIC combo (µg/mL) [b]				
		FLB	MEB	BEN		
AMB	0.25	0.5	0.5	0.5		
FLC	4	4	4	4		
ITZ	0.5	0.25	0.25	0.5		
VOR	0.25	0.125	0.125	0.125		
5FC	8	4	8	8		
**Drug B**	**MIC alone (µg/mL) [a]**	**MIC combo (µg/mL) [c]**				
		**AMB**	**FLC**	**ITZ**	**VOR**	**5FC**
FLB	0.078125	0.078125	0.078125	0.078125	0.039063	0.039063
MEB	0.15625	0.078125	0.078125	0.078125	0.039063	0.078125
BEN	20	5	5	5	2.5	5
